# The primacy of ocular perception: a narrative review on the role of gender identity in eating disorders

**DOI:** 10.1007/s40519-023-01632-6

**Published:** 2024-01-13

**Authors:** Livio Tarchi, Giovanni Stanghellini, Valdo Ricca, Giovanni Castellini

**Affiliations:** 1 Department of Health Sciences, University of Florence, AOU Careggi, Viale della maternità Padiglione 8B, 50126 Firenze, FI Italy; 2 Centro de Estudios de Fenomenologia y Psiquiatria, Universidad ‘Diego Portales’, Santiago, Chile

**Keywords:** Eating and feeding disorders, Embodiment, Gender identity, Socio-cultural factors, Psychopathology, Phenomenology

## Abstract

**Background:**

Phenomenological research has enriched the scientific and clinical understanding of Eating Disorders (ED), describing the significant role played by disorders of embodiment in shaping the lived experience of patients with ED. According to the phenomenological perspective, disorders of embodiment in ED are associated with feelings of alienation from one’s own body, determining an excessive concern for external appearance as a form of dysfunctional coping. The purpose of the present narrative review is to address the role of gender identity as a risk factor for EDs in the light of phenomenological approaches.

**Methods:**

Narrative review.

**Results:**

The current study discusses the interplay between perception, gender identity, and embodiment, all posited to influence eating psychopathology. Internalized concerns for body appearance are described as potentially associated with self-objectification. Furthermore, concerns on body appearance are discussed in relation to gendered social expectations. The current review also explores how societal norms and gender stereotypes can contribute to dysfunctional self-identification with external appearances, particularly through an excessive focus on the optical dimension. The socio-cultural perspective on gender identity was considered as a further explanation of the lived experience of individuals with ED.

**Conclusions:**

By acknowledging the interplay between these factors, clinicians and researchers can gain a deeper understanding of these disorders and develop more effective interventions for affected individuals.

**Level of evidence:**

Level V narrative review.

## Introduction

Eating disorders (ED) are defined, according to the current edition of the Diagnostic and Statistical Manual of Mental Disorders (DSM-5-TR) [1], by specific disturbances in eating behaviors, and by a persistent and undue influence of body weight or shape on the self-evaluation of the individual [1]. Rather than completely demarcated clinical entities, Anorexia Nervosa (AN), Bulimia Nervosa (BN), and Binge Eating Disorder (BED) may share a common psychopathological core [2–4]. In fact, potential transitions between diagnoses have been shown to occur in patient during their lifetime [5–7].

Psychological treatments currently adopted for EDs—such as Cognitive Behavioral Therapy—posit the existence of a specific core of pathological beliefs shared across AN, BN and BED. This psychopathological core is represented by a primary low self-esteem, an over-evaluation of achievements, and a clinically relevant intolerance for adverse mood states, all driving the persistent and undue influence of body weight or shape on self-evaluation [2, 3]. Phenomenological research has offered a novel perspective on this point, focusing on the role played by embodiment in shaping eating psychopathology [8–10].

**Fig. 1:**
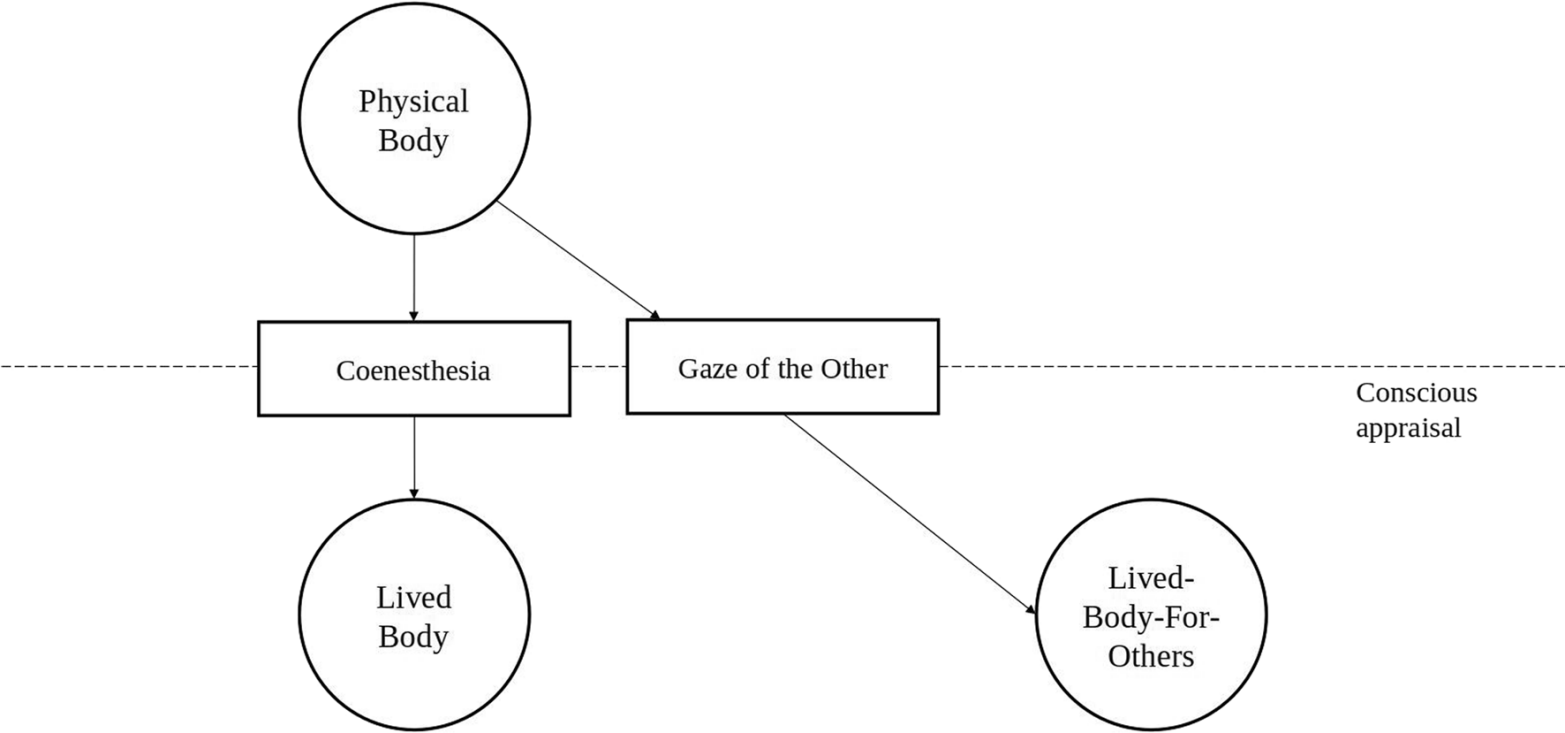
Conceptual summary of key phenomenological concepts. The “physical body” (*Koerper*) is the material, third-person view of one’s own body. The “lived body” (*Leib*) represents the lived experience of it. A third dimension can also be described, which is the first-person view of one’s own body when it is looked by the other (“lived-body-for-others”). Coenesthesia is the integration of perceptual stimuli originating from the body, the foundation of one’s conscious appraisal of their own body. The “gaze of the Other” is an external point of view, subjectively experienced. It acts as a “filter” or “mirror”, through which one’s own body is experienced

Traditionally, phenomenology has developed a distinction between “lived body” (*Leib*)^1^ and “physical body” (*Koerper*)^2^. In this perspective, the “lived body” has been defined as the subjective preconscious, *coenesthetic* 3experience of one’s own body, while the “physical body” represents its material dimension [11, 12]. Recently, Stanghellini and colleagues [9] attempted to conceptualize EDs psychopathology in terms of the “lived-body-for-others”—a concept which was first introduced by Sartre [13]. In addition to the previously described dimensions of corporeality, Sartre described that one can apprehend one’s own body from another point of view, as one’s own body when it is looked at by another person [13]. When we are looked by another person, the “lived body” is no longer a direct, first-personal experiential evidence, but it is an entity that exists as viewed from an external perspective. This third-person perspective of oneself is defined as the “gaze of the Other” (Fig. 1).

According to several observations [19–21], individuals with EDs report a particular difficulty in experiencing their own body from within, or, in other words, from the coenesthetic perspective. The dialectic integration between the inputs arising from the “lived body” and those coming from the “physical body” is impaired. Stanghellini and colleagues [9] have hypothesized that people with ED tend to experience their own body first and foremost as an object looked by another person (the above-mentioned “lived-body-for-others”), rather than from a first-person perspective [14]. This core psychopathological feature would explain the main characteristics of EDs, which are represented by the adoption of external, objective measures to define one’s self and for the clinically relevant preoccupation with controlling one’s own body shape and/or weight [9, 14, 15].

In this perspective, symptoms such as severe dieting or obsessive weight-control might represent a dysfunctional coping strategy to manage the feelings of alienation and extraneousness towards one’s own corporeality. Individuals may report a divergence in the degree to which dieting attempts and eating restriction can be applied or followed [4]. Nonetheless, even when impulsiveness and loss of control over eating represent the main features of the clinical presentation—such as in the case of BED—individuals frequently report a reduced possibility to feel their body from within [9], as well as a perceived similarity between their lived experiences and the lived experience of patients with AN or BN [16]. According to Stanghellini et al. [17], people with EDs define themselves reaching external measures. Thus, they perceive their body as an objectified entity to which aesthetic and moral judgments can be applied.

Further discussion should also be reserved for the process of perception itself. Contemporary experimental research, and previous theoretical studies, have questioned the hierarchy of psychological processes shaping individual perception. Sensory stimuli, rather than constituting the object of perception, can represent external constraints for internal representations [18–20]. In fact, according to recent theoretical frameworks (e.g., active inference), sensory stimuli are first integrated at a preconscious level, and only later cognitively appraised [18]. Therefore, a perceptual and preconscious representation of one’s own body relies at the basis of a conscious experience of it [21]. This novel perspective on perception and consciousness has since triggered a wave of innovations in psychological sciences [14, 22, 23]—while open questions remain in the field of EDs [23].

For instance, as previously mentioned, an altered optical, visible and aesthetic self-appraisal of one’s own body has been postulated for ED^4^ [17, 24]. To individuals with ED, their body would principally be given as an object “to be seen”. The “gaze of the Other” would serve as an optical prosthesis to cope with an altered coenesthesia ^3^ and as a device through which persons with ED can define themselves. This hypothesis has been empirically supported [14, 23, 25–28], and embodiment disorders have been shown to play a role as maintain factors in longitudinal studies focusing on ED symptomatology [29]. Nonetheless, this hypothesis does not yet address the significant gender gap observed within EDs and within clinical practice [1]. In addition, the role of socio-cultural factors in influencing both embodiment and psychopathological symptoms has not been fully previously embedded within this conceptual structure.

The present study thus aims at offering a novel perspective on the strong female preponderance for EDs [1], in light of the role played by the “gaze of Other” in eating psychopathology. For these reasons, a brief review is first offered on the role of socio-cultural factors as informing the lived experience of individuals. Subsequently, the role of gender identity in embodiment is discussed. Finally, the complex interaction between embodiment, gender identity, and psychopathology is presented.

## Lived experience and sociocultural factors

The present narrative review suggests a possible integration of the phenomenological approach with some elements of other available theoretical perspectives (e.g., cognitive–behavioral, psycho-dynamic, psychophysiological findings). Phenomenological explorations typically target the lived experience of individuals. The result is a rich and detailed collection of qualitative self-descriptions from patients. In an attempt to better grasp the pathogenesis of ED, it is crucial to shift the attention from abnormal eating behaviors to more complex and subtler psycho(patho)logical features, especially experiential ones.

For instance, caloric restriction and rapid weight loss are both frequently observed among athletes competing in a specific weight-class [30–32]. These behaviors may even reach clinical significance—possibly inducing side-effects, such as amenorrhea—while still not been conceptualized as constituting a psychopathological disorder [30–32], although potentially satisfying all criteria to be diagnosed with AN or BN. By contrast, a core difference in EDs is that eating behaviors transcend their original significance of nutrition, entertainment, pleasure, and social function. Thus, thinness becomes crucial for self-worth, dietary restraint for the need for control, and binge-eating to manage emotions [10]. The personal meanings behind these behaviors might be analyzed in terms of individual history, and in terms of a dialectical interaction with sociocultural factors, which may vary across cultures and history.

## Sociocultural factors and EDs

Some of the pathological connotations attributed to weight/food control by persons with ED are a product of the fashion industry or media [33–37]. Almost two centuries ago (after the industrial revolution) a sober and controlled alimentation became a spread and largely shared value, and a thin body was considered a symbol of efficiency [38, 39]. Across time the image of the ideal body changed, but it constantly mirrored social position and individual worth, with a number of studies documenting the trend of increasing thinness between the 1950s and the 1990s [40, 41]. Contemporary estimates report a prevalence of 0.7% for EDs in Europe, and a rise of around 15% from 1990 to 2019 for this group of diagnoses in the same region [42].

Common risk factors have been identified for AN, BN and BED [43, 44]. One of the strongest factors were observed in relation to gender [43] and cultural acculturation [43, 45]. Sociocultural influences for EDs have also been noted to interest certain professional sectors more specifically. In particular, those exposed to ideals of beauty and self-control, such as ballet dancers [46–48] and fashion models [49]. Moreover, the importance given to thinness, as an expression of power and control over one’s self [50], as well as a means to reach higher social desirability [46, 51], has been observed as influencing the risk to develop EDs during the lifetime. Interestingly, preliminary evidence has also shown that this risk is influenced by a negative assessment of the position reserved to women in family or society [50]. An effective appraisal of lived experiences along and not in contrast to physical and social determinants is, therefore, of primary importance to the advancement of psychiatry, and to our understanding of EDs in particular.

## Gender identity and eating disorders

More than 70 years ago, Simone de Beauvoir published “Le Deuxième Sexe” (The Second Sex; [52]). Its second volume (“L'Expérience Vécue”, The Lived Experience) is a seminal book, which is arguably at the basis of contemporary thought on gender, gender identity and what a feminine gender entails in general [53–55]. The main focus of this second volume is to ponder “What is woman?” [52]. Beauvoir argues that a woman is, by definition, the “Other”:

“(...) humanity is male, and man defines woman not herself, but as relative to him."

Then, how can the “Other” define itself if not through a third-person view? Positing the feminine as the essential “Other”, Beauvoir implies that a woman is objectified, or, in other words, that a woman becomes connoted by passivity and thus alienated from her true self. Agency, and the active possibility for women to autonomously obtain self-representation and self-definition, is undermined [56]. The essential quality of “Other”-ness can also be internalized [57], with distinct expectations for what concerns gender roles within society and with an explicit focus on the visual representations of the self [58].

The higher prevalence of EDs in Western countries [42], where gender equality is higher [59], can then be interpreted in light of the influence exerted by gender stereotypes on social roles [59]. In fact, occupational segregation between genders may be more readily appraised in more egalitarian and developed countries [60–62]. This cultural and social representation of gender stereotypes, in the occupational or educational sector, can partly explain the equality paradox^5^ [59]. A common theoretical framework to explain this paradox is to posit that virtue-signaling or group-affiliation may shape personal identities, driving social roles to become increasingly more divergent between genders [63–71]. A commonly employed example would be relegating professions involving care to the feminine, and technical oriented careers to the masculine [61].

According to the bio-psycho-social model for EDs [43, 44], biological sex has been recognized as a risk factor for development of these disorders, as demonstrated in both clinical and non-clinical samples. A strong female to male ratio for EDs is estimated from population-based studies [42]. In parallel, a higher risk for ED has consistently been observed at the epidemiological level for transgender women in comparison with transgender men [72], irrespective of gender-affirming hormonal therapy or surgical interventions [73]. Transgender women appear to be at a higher risk of being diagnosed with an ED also when accounting eating restraints aimed at modulating hormonal effects on body weight and shape [73], that is when eating restraints are not secondary solely to gender-distress. Therefore, gender, and not solely sex, should also be recognized as influencing eating behaviors. For this reason, a perspective on lived experiences for women should move beyond mainly characterizing biological characteristics in relation to sex as informing the risk to develop an ED during the lifetime.

While genetic and hormonal factors have a role in eating psychopathology [74], the particular onset of most EDs around puberty [1] may also be appreciated beyond mechanistic or reductionist claims of hormonal influences on mental disorders [75]. In fact, a child going through puberty may feel that their body is escaping them, that their body is no longer the clear expression of their individuality [76, 77]. This experience of alienation from one’s own body is also a function of social expectations for what concerns gender identity [78–81]. In other words, female and male adolescents who do not fully conform to gendered expectations for what concerns primary or secondary sexual characteristics, or who do not fully conform to social expectations for what concerns gender roles or gender expression, may be at a higher risk of experiencing feelings of alienation from their body [82–85].

In this instance, the body becomes foreign, and, at the same moment, it is grasped by others as an “object”. If the child is a woman, she may also more frequently be objectified or sexualized [86]. The visual, or ocular characteristics, are those more readily grasped by the “gaze of the Other”, and may thus become a primary target for body modification goals, or concealment [73, 87].

## Gender identity and embodiment

Since ancient times, the optical dimension has been specific to the feminine, and the mirror is the feminine utensil par excellence—at least in the stereotypical and common-sense meaning [88, 89]. It evokes the radiance of beauty, the charm of the gaze, seduction. To reflect oneself in the mirror is to project one's image before oneself, to split oneself into a self that is looked at and one that is looked at. The mirror is used to see, know, modify, and disguise oneself. The face in Greek is called prosopon—the figure that offers itself to the eyes of the “gaze of Other” as a seal of its own identity [90]. Female identity has thus always been linked to the optical dimension—both to one's own appearance reflected in the mirror and to one’s own appearance offered to the “gaze of the Other”.

This dependence of identity on gaze (not female only), and especially on the “gaze of the Other”, has not diminished in the course of history, but on the contrary has been further strengthened in the “society of the spectacle” [91] whose distinctive trait is, precisely, “ocular-centrism” [92]. This mode of access to oneself mediated by visual representations can turn out to be alienating, since images convey individual ghosts and cultural aspects, social prejudices, gender stereotypes. At the same time, the attempt to experience and define one’s own self through the “gaze of the Other” may be captivating or socially rewarding [93]. However, defining one’s self in this manner exposes to the risk of being enthralled into an alienated representation of the self, fully enmeshed and intertwined within social expectations [94], in complete opposition to an authentic definition of the self.

Theoretically, any individual can ultimately succeed in grasping itself only by alienating itself, positing oneself both as a subject and, vis-à-vis oneself, as an object [13]. In fact, the act of defining oneself is not solipsistic in nature, but requires another being to compare oneself, and to which to be compared [95]. Moreover, the “Other”, as an existent being itself, can form a representation of us in its mind, and we, in turn, can define ourselves as a function of this representation [96].

**Fig. 2:**
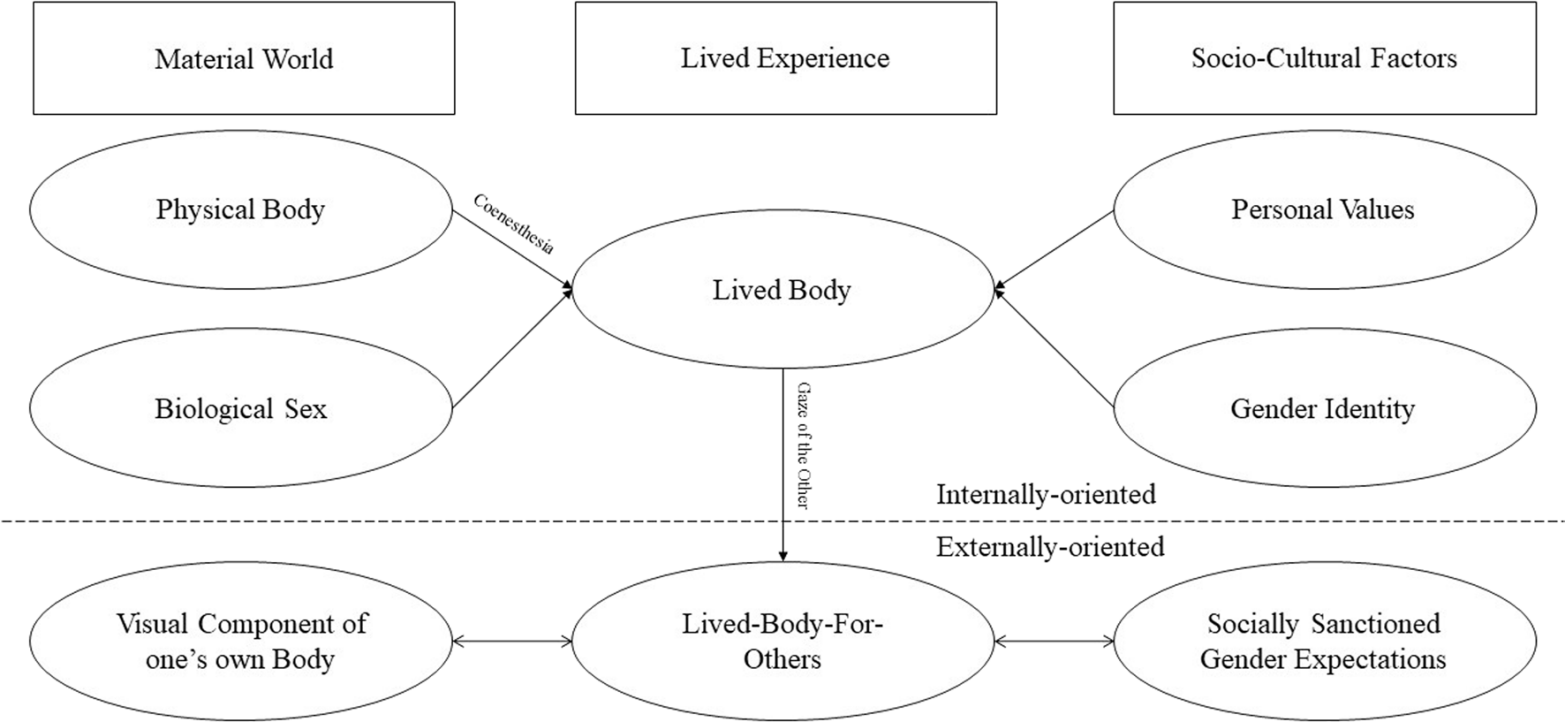
Proposed novel framework of interactions between embodiment and gender identity. The “gaze of the Other” can play a dual role. On one hand, it may exert a violent action, subjugating one’s own visual dimension as the forefront component of the self. On the other, reassuring, offering a cohesive representation of the self. The “lived-body-for-others” can thus become an external validation of one’s own gender identity, or impose socially sanctioned gender expectations, which may be experienced as distressing at the individual level

Women have not been equally supported in the maturation of an autonomous definition of their identity by the presence of widespread, culturally and socially relevant gender models, by which to define themselves [97]. Similarly, gender minorities and non-stereotypical males may experience psychological distress secondary to social expectations in relation to their body weight or shape [98–102], as well as their gender roles or their gender expression [103]. Social expectations for what concerns “feminine” can strongly influence the lived experience of any individual [52, 104], and the essential quality of the “Other” internalized in relation to gender identity (Fig. 2).

## Gender identity, embodiment, and psychopathology

The interplay between gender identity, embodiment and psychopathology has not yet been fully elucidated. Here, the authors posit that the “gaze of the Other” can exert a dual effect on the individual. On one hand, the “gaze of the Other” offers an external reality, capable to offer us self-recognition and validation [105]. On the other, it may be a source of distress, defining us, possibly in contrasts with our personal values [13].

The individual may react to the challenge posited by the “gaze of the Other” symmetrically and in a dual manner: either rejecting this external “definition” of itself, or, vice-versa, seeking it, to possibly grasp itself through a well-defined, stable and “objectified” representation [106–108]. A healthy well-being may result from the balance of these two processes. On the contrary, a maladaptive self-identification can be attempted in persons with ED [23], fueled by a disproportion in the preconscious optical–coenesthetic experience of oneself. Nonetheless, the attempt to nullify oneself to resist an external definition may be observed within EDs as well. As the visible body is the channel by which oneself is subjugated by the “Other”, coercing it can diminish the possibility of being grasped. This process is more readily apparent in victims of abuse or sexual violence [27, 28, 109]. Nullifying one’s own body can represent a form of self-injury and self-preservation at the same time [110].

Repetition of trauma and hypersexuality may also be observed, even in individuals with a history of adverse experiences in the sexual domain [111–114]. In fact, while previous theoretical contributions focused on the role of trauma in predisposing individuals for emotional dysregulation, and thus, potentially, hypersexuality, sexual activity can also represent an attempt to alienate and subjugate oneself in a perceptual manner [111]. In addition, it may reflect an effort to experientially, affectively, seek an alternative encounter with one’s own body, thus escaping a dysfunctional optico-coenaesthesis^6^ [115].

## Strengths and limits

The strength of the current study lies in its phenomenological approach. The subjective experience of individuals with ED was considered, which allowed for a deeper and nuanced understanding of their lived corporeality. Previous theoretical contributions have been integrated with a sociocultural perspective, in light of self-objectification theory. A novel perspective on the gender gap observed within AN, BN and BED was reached. In summary, the current study proposes the interplay between perception, gender identity and embodiment as a potential target for future empirical research and clinical interventions.

The limits of the study, on the other hand, are represented by its narrative nature, relying on the existing literature. Furthermore, while the study discusses important theoretical implications of gender identity and perception on embodiment, it may lack full empirical evidence to support these claims, and its exploratory intent should be taken into consideration.

## What is already known on this subject?

Some key features of embodiment disturbances in ED have been previously described, such as the experience of a distorted body image, or the lack of effective integration between interoceptive or visual stimuli within an appropriate cognitive appraisal of body weight and shape. In addition, individuals with ED report feelings of alienation from their own body, feeling their material self to be extraneous from themselves. For this reason, a subjective experience of ‘estrangement’ from their physical self is known to fuel distress in these individuals, and thus contribute to disordered eating patterns. As a response, individuals with ED frequently report relying on external measures to reach an effective definition of their corporeality.

## What this study adds?

The present narrative review attempts to integrate phenomenological accounts with the psychosocial model for EDs, remarking the role of the broader social context in which these disorders develop and are experienced. Accordingly, the role of the feminine as the “Other” was discussed as shaping self-objectification among women, and as a partial explanation of gender discrepancies in the epidemiology of EDs. The current review also emphasizes the need to move beyond solely describing biological sex as contributing to the female/male disparity in EDs, advocating for a socio-cultural perspective on gender identity.

## Conclusions

As long as the “feminine” is lived and conceptualized as the “Other”, the feminine gender will be associated with a higher risk to adopt a dysfunctional identification of the self through the “gaze of the Other”, especially in an ocular-centric society. Current theoretical models positing a role for embodiment in shaping eating psychopathology can be updated, appreciating the interplay between the feminine gender and the risk to engage in maladaptive self-objectification. This maladaptive self-objectification may be attempted through the subjugation to the visual representation of oneself through the “gaze of the Other”. The interplay between gender and EDs needs to be considered as embedded within a world of values—both at the individual and sociocultural level.

## Data Availability

Not applicable.

## Footnotes

1 One’s body experienced *from within*, i.e. from a first-person perspective. Also called the *body-I-am* or body-subject.

2 One’s body apprehended *from without*, i.e. from a third-person perspective. Also called the *body-I-have* or body-object.

3 The overall collection of perceptual stimuli originating from the body, which creates the foundation for one's consciousness of their own body or their own physical condition, such as the sense of well-being, energy, or alertness.

4 *The Optical-Coenesthetic Disproportion Hypothesis*. Under normal conditions, the apprehension of one’s body through coenesthesia and through the other’s look are in a dynamic balance. The *optical-coenaesthetic proportion* is a prerequisite for constructing a dependable sense of bodily self and personal identity. In persons with ED, this dialectic breaks down. Particularly relevant to understanding a person with ED is to envision in the “gaze of the Other” a kind of visual prosthesis that helps him/her feel his/her own body.

5 That is, the paradox that specific gender biases, such as the under-representation of women in math-related fields, seems more pronounced in more developed countries.

6 As in, the adoption of an optically-driven definition of one’s self, given the altered experience of one’s own body from within—the coenaesthetic perspective.
